# On Your Feet to Earn Your Seat: pilot RCT of a theory-based sedentary behaviour reduction intervention for older adults

**DOI:** 10.1186/s40814-017-0139-6

**Published:** 2017-05-08

**Authors:** Isabelle White, Lee Smith, Daniel Aggio, Sahana Shankar, Saima Begum, Raluca Matei, Kenneth R. Fox, Mark Hamer, Steve Iliffe, Barbara J. Jefferis, Nick Tyler, Benjamin Gardner

**Affiliations:** 1 Health Behaviour Research Centre, Department of Epidemiology and Public Health, University College London, London, UK; 2 The Cambridge Centre for Sport and Exercise Sciences, Department of Life Sciences, Anglia Ruskin University, Cambridge, UK; 3 Research Department of Primary Care and Population Health, University College London, London, UK; 4 Department of Psychology, Institute of Psychiatry, Psychology and Neuroscience, King’s College London, De Crespigny Park, London, SE5 8AF UK; 5 Centre for Music Performance Research, Royal Northern College of Music, Manchester, UK; 6 Centre for Exercise, Nutrition and Health Sciences, University of Bristol, Bristol, UK; 7 National Centre for Sport and Exercise Medicine, Loughborough University, Loughborough, UK; 8 Population Health Domain Physical Activity Research Group, Department of Epidemiology and Public Health, University College London, London, UK; 9 UCL Department of Civil, Environmental and Geomatic Engineering, University College London, London, UK

**Keywords:** Older adults, Sedentary behaviour, Sitting, Physical activity, Intervention, Habit, Behaviour change

## Abstract

**Background:**

Of all age groups, older adults spend most of the time sitting and are least physically active. This sequential, mixed-methods feasibility study used a randomised controlled trial design to assess methods for trialling a habit-based intervention to displace older adults’ sedentary behaviour with light activity and explore impact on behavioural outcomes.

**Methods:**

Eligibility criteria were age 60–74 years, retired, and ≥6 h/day leisure sitting. Data were collected across four sites in England. The intervention comprised a booklet outlining 15 ‘tips’ for disrupting sedentary habits and integrating activity habits into normally inactive settings, and eight weekly self-monitoring sheets. The control was a non-habit-based factsheet promoting activity and sedentary reduction. A computer-generated 1:1 block-randomisation schedule was used, with participants blinded to allocation. Participants self-reported sedentary behaviour (two indices), sedentary habit, physical activity (walking, moderate, vigorous activity) and activity habit, at pre-treatment baseline, 8- and 12-week follow-ups and were interviewed at 12 weeks. Primary feasibility outcomes were attrition, adverse events and intervention adherence. The secondary outcome was behavioural change.

**Results:**

Of 104 participants consented, 103 were randomised (intervention *N* = 52, control *N* = 51). Of 98 receiving allocated treatment, 91 (93%; intervention *N* = 45; control *N* = 46) completed the trial. One related adverse event was reported in the intervention group. Mean per-tip adherence across 7 weeks was ≥50% for 9/15 tips. Qualitative data suggested acceptability of procedures, and, particularly among intervention recipients, the allocated treatment. Both groups appeared to reduce sedentary behaviour and increase their physical activity, but there were no apparent differences between groups in the extent of change.

**Conclusions:**

Trial methods were acceptable and feasible, but the intervention conferred no apparent advantage over control, though it was not trialled among the most sedentary and inactive population for whom it was developed. Further development of the intervention may be necessary prior to a large-scale definitive trial. One possible refinement would combine elements of the intervention with an informational approach to enhance effectiveness.

**Trial registration:**

ISRCTN47901994 (registration date: 16th January 2014; trial end date 30th April 2015)

**Electronic supplementary material:**

The online version of this article (doi:10.1186/s40814-017-0139-6) contains supplementary material, which is available to authorized users.

## Background

While the benefits of physical activity (PA) for health are well-documented [1], an emerging literature suggests that sedentary behaviour (SB)—i.e. actions undertaken while sitting or reclining that expend 1.5 metabolic equivalents or less [2]—represents a potentially independent risk factor for mortality and morbidity [3–5]. It has been suggested that prolonged SB may be offset by around an hour of daily moderate-to-vigorous PA [6], but given the high prevalence of SB and low prevalence of PA among the general public [7], this may be an unrealistic behavioural target for many. Sedentary and inactive lifestyle puts older adults at particular risk; of all age groups, people aged 60 or older spend most waking hours sitting and do least PA [8–11]. Interventions are needed to reduce SB in older adults, ideally by displacing sitting time with light or more intensive PA [12–15].

Few SB-reduction interventions have been developed for older adults. Several studies have variously reported reductions in SB indices or increases in light and moderate PA following provision of accelerometer feedback to older adults and individualised consultations on modifying SB [16–19]. Self-regulatory strategies—e.g., setting goals, providing normative feedback, problem-solving and planning—have also been associated with declines in SB among older adults [18, 20, 21]. However, these interventions have been evaluated using uncontrolled, pre-post designs. Moreover, these proposed intervention strategies have typically involved provision of one-to-one behavioural support. Such strategies are not only relatively resource-intensive, but also risk yielding only short-term benefits, which dissipate when intervention delivery ceases.

Habit formation has been proposed as a route to self-sustained behaviour change [22]. Making PA habitual—i.e. automatically triggered in specific contexts, due to learned associations between contextual cues and actions [23, 24]—may ‘lock in’ PA gains over time [22, 25]. Habits develop through ‘context-dependent repetition’; repeatedly performing an action in a particular context reinforces context-behaviour associations, such that the habitual response becomes dominant in memory [26]. As habit forms, control over initiation of action becomes less reliant on memory, attention and motivation, making the behaviour automatic and easier to perform [27]. By virtue of its automaticity, habitual PA may be performed even when conscious intentions are weak [28]. Tentative evidence suggests that simple actions may become habitual more quickly than complex actions [29]. Integration of ‘small’ PA that changes into everyday routines (such as balance exercises while working at a kitchen bench), may be the most feasible strategy for forming habits, and so maintaining behaviour, among sedentary and inactive older adults [30–32].

### The present study: aims and objectives

This study presents a pilot trial of an intervention, based on the habit-formation model, which aims to reduce and displace SB with light PA among older adults [33]. The intervention centres on a booklet (titled ‘On Your Feet to Earn Your Seat’), comprising tips for reducing sitting and integrating PA habits into everyday routines, and a series of tick-sheets to self-monitor progress. Our previous uncontrolled trial, undertaken in two samples of older adults aged 60–75 years, demonstrated that both samples viewed the intervention positively, found the tips easy to follow and reported health and wellbeing improvements [34]. This paper reports findings from a sequential, mixed-methods feasibility study consisting of a parallel randomised controlled trial, comparing our intervention to a non-habit, information-only control treatment and subsequent semi-structured interviews with trial participants. The study was designed to inform a decision about whether to proceed to a large-scale definitive controlled trial and had two objectives: first, to explore the feasibility of trial procedures and acceptability of the allocated treatments and second, to explore potential effects on sedentary and PA behaviour and habit. We intended to progress to a larger trial if trial procedures were feasible, and the intervention acceptable.

The present trial is registered (ISRCTN47901994). Finer theoretical rationale, methodological details and unforeseen deviations from registered procedures have been described in an open-access published protocol [33, 35].

## Methods

### Study design and procedure

This study used a sequential mixed-methods design, consisting of a RCT that generated quantitative data and subsequent semi-structured interviews that generated qualitative data on participants’ experiences of the allocated treatment and trial procedures more broadly. A parallel two-arm RCT was undertaken, with participants individually randomised to receive either the habit-based intervention (intervention group) or a pre-existing fact-sheet describing UK government recommendations for PA and SB in older adulthood ([36]; control group). Participants were recruited from one of four clusters of sites in England: two general practices in north London; a foundation trust in Lincolnshire (Lincs); the outpatients’ department of a general hospital in Surrey; and three general practices in Kent. Procedures were tailored according to resources at each site and were conducted by a team local to each site. All teams were trained by the Chief Investigator (BG; a social and health psychology researcher) and received a manual of localised study procedures. Data collection at the London sites was conducted by a team comprising a post-doctoral practising health psychologist and four Masters-level graduate students of health psychology. Data collection at all other sites was conducted by a local research team of clinical studies officers with trials administration expertise, but no prior psychology or behaviour change qualifications.

At all sites, recruitment took place between June 2014 and January 2015, and data were collected between June 2014 and April 2015. Data collection was pre-planned to end in April 2015 due to funding constraints. While we sought a sample of 120 participants (60 per treatment group) to obtain sufficient data to capture variability and to inform a power calculation for a definitive RCT [33], no sample size limits were imposed at any site. Each site recruited as many participants as possible within the study period.

Potential participants were identified via a mailout at London, Surrey and Kent sites and public advertisements at the Lincs sites. Self-reportedly eligible individuals were consented into the study in their home or at a research clinic. Participants were randomised, after consent, by an independent trial administrator, using a computer-generated 1:1 block randomisation schedule [37]. Subsequent procedures were administered at research visits in participants’ homes (London, Lincs), a research clinic (Surrey) or the home or research clinic according to preference (Kent). Participants were blinded to allocation, but data collectors and outcome assessors were not. Due to resource constraints, we neither assessed nor planned to assess intervention adherence or fidelity.

The allocated treatment was delivered around 1 week post-consent (i.e. the baseline visit). Participants completed self-report measures of behaviour, health and wellbeing prior to each of three research visits (baseline, 8 and 12 weeks post-baseline). The questionnaire was collected and objective functioning measures taken at each visit. A semi-structured exit interview was conducted at 12 weeks. At the London site only, participants received a £10 shopping voucher at each visit, and an additional £30 voucher conditional on completing all visits.

Primary outcomes focused on feasibility and acceptability (attrition, adverse events and, among the intervention group, adherence). Secondary outcomes were changes in behaviour (PA and sitting behaviour and habit). We also recorded, via interviews, participants’ experiences of the study, to provide qualitative data to complement, elucidate and expand on findings from quantitative analyses. All procedures were approved by an NHS Research Ethics Committee (ref 13/LO/1549) and Clinical Research Networks local to each site.

### Participants

Eligible participants were: aged 60 to 74 years^1^, self-reportedly retired and sedentary (≥6 total leisure hours sitting per day). People with physical impairments precluding light intensity PA, lacking capacity to provide informed consent, living in the same household as another study participant, or unable to speak or read English fluently were ineligible. A planned inactivity criterion (self-reported ≤30 consecutive minutes of leisure time physical activity of ≥3 metabolic equivalents per week) was removed at the early stages of the trial because all participants meeting the criterion at consent were found to have increased their activity above this level at baseline [35]. Due to errors made by a commercial mailout company and the postal service^2^ (see [35]), recruitment rates (i.e. proportion of respondents to mailouts at London and Kent sites) could not be reliably estimated.

### Intervention and control treatments

The intervention consisted of a printed A5-sized information booklet outlining the health impact of SB and PA and 15 tips on reducing SB and forming PA habits, with eight printed ‘tick-sheets’ for participants to record daily adherence to tips for both intervention and data collection purposes. Tips recommended light PA (i.e. activity within the range of 1.5–3.0 metabolic equivalents [15]), covering aerobic, balance, flexibility, and muscle-strengthening exercises, and reducing SB. Where possible, tips specified an everyday cue (e.g. ‘when standing by the kitchen sink…’) and a behaviour for performance when encountering the cue (‘…stand on your tip toes and drop back down onto your heels’), with a health-related rationale (‘this will increase bone density and reduce likelihood of falls’). ‘Handy hints’ were provided to offer less or more intensive variants of proposed activities, or actions likely to increase enactment. We originally planned to offer the intervention group, at 4 weeks post-baseline, motivational phone support from a practising health psychologist, but did not do so because we deemed it unfeasible for non-London site teams to be adequately trained in offering personalised, responsive and evidence-based behaviour change advice. A comprehensive description of intervention content is provided in Additional file 1: Table S1.

The control group received a printed A4-sized one-page NHS factsheet that outlines the health consequences of PA and SB, and describes UK government recommendations for the duration, frequency and intensity of PA, and suggests that sedentary time is minimised. It also provides examples of activities that increase PA and suggestions for reducing SB (see [38]). Both treatments were administered face-to-face in an individual session with each participant at the baseline visit.

### Data collection

Unless indicated, at all sites all data were collected at baseline, 8 and 12 weeks.

#### Demographics

Sex, age, ethnicity and education level (age when leaving school; university attendance [yes/no]) were self-reported in the baseline questionnaire.

#### Primary outcomes: acceptability and feasibility

All sites were required to immediately notify the Chief Investigator of adverse events using a pro forma. Adherence to tips among the intervention group was calculated from seven tick-sheets for which full data were available (Weeks 2–8), each tick indicating that the participant had completed a corresponding tip on a specified day. Semi-structured interviews at 12 weeks focused on motivation for participation, and experiences of study procedures and allocated treatments, and were digitally recorded and transcribed verbatim. The duration of each research visit was also recorded, using a stopwatch, but these data are outside of the scope of this paper so are presented as supplementary material.

#### Secondary outcomes: behaviour and habit

##### Behaviour

Total PA and SB were self-reported using the short-form International Physical Activity Questionnaire (IPAQ [39]), a measure that has shown test-retest reliability and convergence with objective PA and SB indices [39, 40], and the Measure of Older Adults’ Sedentary Time (MOST [41]), which has been validated against objectively measured inactivity and summarises sedentary behaviour across seven domains (e.g. watching television, reading). Both measures operationalise SB as sitting time, and were adapted to refer to activity on the preceding day, to aid recall accuracy. IPAQ measures captured sitting, walking, moderate and vigorous PA. The latter three were measured via two items: ‘Did you do any [walking/moderate physical activities/vigorous physical activities] yesterday?’ (yes/no), ‘(If yes:) How much time did you spend walking/doing moderate physical activities/vigorous physical activities] yesterday?’ SB was captured by a single item (‘How much time did you spend sitting yesterday?’). Moderate PA was defined as ‘activities that take moderate physical effort and make you breathe somewhat harder than normal’, and vigorous PA ‘activities that take hard physical effort and make you breathe much harder than normal’. Responses to all IPAQ and MOST items were provided in hours and minutes and were converted to minutes for analysis purposes. MOST data were summed across the seven activities to produce an aggregate score.

At the London sites only, participants were fitted with a thigh-worn accelerometer-inclinometer device (activPAL; PAL Technologies, Glasgow, Scotland) for 7-day wear, 1 week prior to baseline, 8- and 12-week visits, to capture SB and PA objectively. activPAL devices are posture-sensitive, so distinguish sitting (a form of SB) from standing or other light PA [42]. In the intervention group only, adherence to tips up to 8 weeks was self-reported using 8 × 7-day tick-sheets, with a tick recorded on each day a recommended activity was performed at least once.

##### Habit

SB (i.e. sitting) and PA habit strength were each measured via a single item derived from the Self-Report Habit Index [43], previously validated to capture automaticity (i.e. ‘[Sitting/physical activity] is something I do without thinking’ [44]).

##### Health, physical functioning and wellbeing

**Fig. 1:**
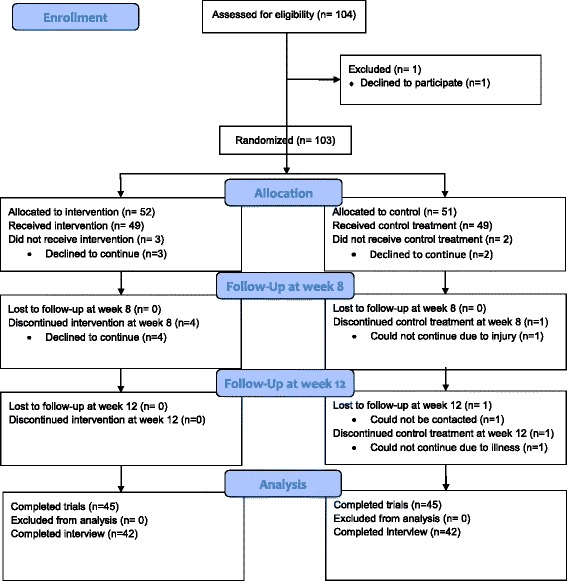
CONSORT flow diagram

Measures of objective physical functioning, and self-reported health and wellbeing, were also taken [33], but are outside of the scope of this paper. Descriptions and analyses of these are presented as supplementary material (Fig. 1).

### Progression criteria

Data were evaluated according to whether they met the following criteria for progression from the present study to a larger, definitive trial: no serious adverse events (i.e. hospitalisation, life-threatening, death) occurred, and any adverse events related to treatment were experienced by less than 5% of participants in either group (~3 participants per group); attrition in either group was below 17%; and intervention adherence was above 50%. An additional criterion was that apparent between-group differences, regardless of statistical significance, should point to substantial gains on more behavioural indices among the intervention group relative to control. If all criteria were met, we intended to progress directly to a definitive trial. If the three former criteria were met and the additional criterion is not met, we intended to further refine the intervention prior to a definitive trial. If any of the former three criteria were not met, we intended to further refine the trial methods prior to any definitive trial.

No adverse events were anticipated in either group, given the emphasis of the intervention on incremental gains in light PA, and that the control treatment was publicly available. The 17% attrition criterion was based on an omnibus attrition rate derived from a review of 22 previous PA-promotion interventions (albeit over a 6-month period [45]). Although not directly related to trial feasibility, behavioural outcomes were used to inform a decision on whether the intervention was fully ready for a definitive trial.

### Analysis

#### Primary outcomes: acceptability and feasibility

Attrition rates were summarised using descriptive statistics. Demographics, SB and PA for study completers and non-completers are described in Additional file 2: Table S2, and research visit duration across sites described in Additional file 3: Table S3.

Three adherence metrics were derived (Additional file 4: Table S4). *Mean weekly adherence* to each tip was computed by summing total ticks recorded for that tip in that week and dividing by seven (i.e. 7 days). Mean total adherence to each tip was computed by summing all ticks for that tip across all seven tick-sheets and dividing by 49 (i.e. 7 days × 7 weeks). A single *global mean total adherence* score was calculated by summing mean total adherence to each of the 15 tips and dividing by 15. All rates were multiplied by 100, for expression as percentages. Adherence was deemed to meet our progression criteria where the global mean total adherence score was above 50%.

Experiences of participation were synthesised via thematic analysis of verbatim interview transcripts. Due to limited resources, only a randomly selected subset of interviews were coded. We compensated for the lower quality of non-London site interviews, which could often not be extensively coded, by coding all 21 London site interviews. Of 84 interviews conducted, the analytic dataset thus comprised 46 interviews (55% of available data; 24 intervention, 22 control), including five each from Lincs and Surrey, and five from each of the three Kent subsites. Although not all interviews were analysed, theoretical saturation was reached from the London interviews alone, with no significant new findings emerging from interviews from non-London sites.

#### Secondary outcomes: behaviour and habit

To assess the validity of self-reported behaviour, Spearman correlations, which allow for non-normally distributed data, were calculated between objective and self-reported SB and PA, using data from the London sites (Additional file 5: Table S5). Prior to analysis, raw activPAL data were visually inspected for unusual episodes. Participants for whom SB of 22 h or more was recorded on a single day in one of the 3 monitoring periods were assumed to have not worn the device for 7 full days and were removed from analyses pertaining to that monitoring period. For all other participants, SB data were derived from a standardised 16 h period (7 am–11 pm).

Given the exploratory nature of analysis, changes over the 12-week study period in behaviour and habit (and health and wellbeing; Additional file 6: Table S6) were visually inspected. Inferential statistical tests were not employed, because the sample was not a priori powered to formally assess intervention effectiveness. To account for non-completers, corresponding data using baseline-observation-carried-forward analyses are reported in Additional file 7: Table S7. To assess whether behaviour change might be attributable to habit formation, correlations were calculated between changes in SB and PA frequency and habit strength (Additional file 8: Table S8).

## Results

### Sample description

Participants were consented between 26th June 2014 and 29th January 2015, with the final follow-up conducted on 30th April 2015. Across the four sites, 104 eligible participants were consented. Six withdrew prior to baseline. Of the remaining 98 participants, 49 were allocated to intervention and 49 to control. Around half of the sample was from the Kent site (*N* = 46).

At baseline, SB estimates were consistently lower using the IPAQ than the MOST measure. On both measures, standard deviations indicated that estimated daily SB at baseline (typically 1 week post-consent) was below the 6 h/day (360 min/day) entry level criterion for a considerable proportion of participants in both groups, suggesting that SB levels may have changed between consent and baseline measurement. Consenting to participate may have stimulated SB reduction prior to treatment allocation.

Three themes emerged from the qualitative data. Two of these related to the acceptability of trial procedures and the allocated treatments, so are presented alongside primary outcomes below. One related to perceived behavioural and health changes and is presented alongside secondary outcomes (Table 1).

**Table 1:** Baseline demographics, physical activity and sedentary behaviour, including completers and post-baseline non-completers

Characteristics		All participants	Intervention group (*N* = 49)	Control group (*N* = 49)
Demographics				
Gender: *N* (%)	Available, *N*	97	48	49
	Female	57 (59%)	29 (60%)	28 (57%)
Age (years)	Available, *N*	95	46	49
	Mean (SD)	68.32 (3.78)	68.00 (4.05)	68.61 (3.52)
Ethnicity: *N* (%)	Available, *N*	94	46	48
	White	91 (97%)	44 (6%)	47 (98%)
	Black	0	0	0
	Asian	1 (1%)	1 (2%)	0
	Mixed or other	2 (2%)	1 (2%)	1 (2%)
Marital status: *N* (%)	Available, *N*	96	47	49
	Single	7 (7%)	6 (13%)	1 (2%)
	Married	72 (75%)	35 (75%)	37 (76%)
	Widowed	9 (9%)	2 (4%)	7 (14%)
	Divorced or separated	8 (8%)	4 (9%)	4 (8%)
Longstanding illness: *N* (%)	Available *N*	94	46	48
	Yes	69 (73%)	38 (83%)	31 (65%)
Education, university: *N* (%)	Available *N*	92	46	46
	Yes	33 (36%)	14 (30%)	19 (41%)
Education, age leaving school (years)	Available *N*	91	45	46
	Mean (SD)	16.34 (1.38)	16.11 (1.47)	16.57 (1.28)
Sedentary behaviour				
Sitting time (IPAQ; min/day)	Available, *N*	90	45	45
	Mean (SD)	480.30	483.60 (212.25)	477.00 (159.99)
Sitting time (MOST; min/day)	Available, *N*	96	48	48
	Mean (SD)	559.34 (213.99)	552.54 (206.18)	566.15 (223.49)
SB habit^a^	Available, *N*	95	47	48
	Mean (SD)	4.13 (0.80)	4.11 (0.89)	4.15 (0.71)
Physical activity				
Walking (min/day)	Available, *N*	90	46	44
	Mean (SD)	94.50 (90.62)	77.61 (67.80)	112.16 (107.52)
Moderate PA (min/day)	Available, *N*	95	47	48
	Mean (SD)	33.95 (60.85)	36.81 (60.42)	31.15 (61.77)
Vigorous PA (min/day)	Available, *N*	96	47	49
	Mean (SD)	9.64 (30.63)	8.19 (26.24)	11.02 (34.54)
PA habit^a^	Available, *N*	95	47	48
	Mean (SD)	3.27 (1.13)	3.30 (1.21)	3.25 (1.06)

^a^Habit measured on a 1–5 scale, where *1*, weak or no habit and *5*, strongest habit

### Primary outcomes: acceptability and feasibility

#### Attrition and adverse events

Of those receiving the allocated treatment (*N* = 98), 45/49 (92%) intervention and 46/49 (94%) control participants completed the 12-week trial. Four intervention participants discontinued at 8 weeks, for reasons not reported. One control participant discontinued at 8 weeks (injury unrelated to participation), and two at week 12 (one due to illness unrelated to participation and one could not be contacted). Attrition in both groups was thus below the 17% criterion, so was deemed satisfactory. Attrition rates did not appear to differ between groups, nor were there differences between trial completers and non-completers (Additional file 2: Table S2). Of 22 participants at the London site, all were fitted with accelerometers at all three time points, with 21 (95%) completing the 7-day accelerometer wear protocol at baseline, 19 (86%) at 8-week and 19 (86%) at 12-week follow-up.

One adverse event occurred: an intervention recipient reported that her shoulder became stiff and painful when attempting a stretch exercise recommended within the intervention booklet (tip not specified). She received GP and physiotherapist support and continued to try to adhere to the tips, completing the 12-week trial. No serious adverse events were recorded, so both treatments were deemed satisfactory.

#### Adherence to tips (intervention group only)

Of 49 intervention group participants, 39 (80%) returned completed tick-sheets for analysis. Global mean total adherence, across all tips and all weeks (52.50%; 95% CI: 44.42, 60.48), was above our 50% cut-off and so the intervention was deemed satisfactory. Highest mean total adherence, across all weeks, was observed for tip 1 (‘leave the house daily’; 74.46% [65.41, 82.83]), and lowest for tip 8 (‘improve your posture’; 31.71% [20.46, 44.16]). Mean total adherence was above 50% for 9 of the 15 tips, indicating that these were more often performed than not (Table 2). Per-tip mean weekly adherence rates were consistently lowest at week 8, but the weeks at which adherence peaked varied (Additional file 4: Table S4).

**Table 2:** Mean total adherence to each tip (*N* = 39, intervention group only)

Tips	Mean total adherence (95% CI)	Range of mean weekly adherence rates
1. ‘Leave the house daily: Ensure that you go out at least once a day.’	74.46% (65.41, 82.83)	65.57% (week 8 [W8])–79.85% (W3)
2. ‘Make ad breaks active: When you watch TV, stand up or walk around during breaks between programmes.’	49.92% (37.31, 61.85)	42.12% (W8)–58.97% (W2)
3. ‘Take a stand: Stand up when waiting for a bus or train.’	52.07% (41.03, 64.05)	38.46% (W8)–58.24% (W2)
4. ‘Time to stretch: When sitting for long periods … set an alarm to go off every 20 minutes. When it rings, stand up and stretch … as high up as you can at least five times.’	48.72% (37.15, 59.92)	41.76% (W8)–55.68% (W4)
5. ‘Rising and sinking: When standing by the sink in the kitchen … stand on your tip toes and then slowly drop back down onto your heels. Do this five times, building up to 30.’	63.27% (52.23, 73.26)	50.92% (W8)–72.53% (W3)
6. ‘Watch your step: Try to do at least 30 minutes of walking in total over the course of the day.’	59.45% (48.46, 69.91)	54.58% (W8)–64.84% (W7)
7. ‘Sit to stand with no hands: Each time you stand up, try doing it without using your hands.’	67.40% (56.46, 78.23)	49.08% (W8)–69.96% (W2)
8. ‘Improve your posture: Stand with your back to the wall with your heels two inches from it … and move the back of your head towards the wall.’	31.71% (20.46, 44.16)	28.57% (W3)–35.16% (W8)
9. ‘Limber up:		
9a. Calf stretch	57.46% (44.91, 69.44)	53.11% (W8)–60.81% (W4)
9b. Chest stretch	53.38% (41.13, 65.56)	46.89% (W8)–57.51% (W3)
9c. Walk as if on a tightrope across the floor	39.67% (28.10, 51.23)	32.97% (W8)–45.42% (W6)
9d. March on the spot	56.88% (46.78, 68.97)	52.01% (W2)–63.00% (W6)
9e. Walk your fingers up the wall	38.51% (27.16, 50.13)	35.90% (W7)–41.39% (W6)
9f. Lift a tin of food in each hand.’	39.19% (27.37, 52.85)	35.16% (W2)–43.59% (W6)
10. ‘Wall push-ups: do 10-push ups against a wall each morning.’	55.42% (44.11, 66.82)	49.08% (W8)–61.54% (W6)

*SD* standard deviation, *W* week number. Observed range for all tips: 0–100%.

#### Qualitative analysis

##### Acceptability of trial procedures

Participants indicated that they were generally motivated to participate to gain feedback on or improve their health and fitness, though some participated to express support for the research team or general practice that recruited them. There was no indication that shopping vouchers (London sites) incentivised participation.

No participant reported objecting to research visits, with most reporting them to be convenient and enjoyable. Those who wore accelerometers generally initially found them odd, but they became unobtrusive (‘I put them on and just forgot about them’; London, control, participant ID202). Some reported mild skin irritation from the adhesives, so they declined to wear the device for the prescribed period.

Some questionnaire items were deemed difficult to complete, especially recalling SB over the previous day (‘I looked over the week and said, how long, typically, rather than on average, do I spend doing each of those [seated activities]?’; London, control, ID241). Some questions were deemed too restrictive to elicit meaningful responses. Some felt the objective measures were time-consuming (‘I’ve really enjoyed having my blood pressure taken 200 times’; Kent, control, ID28).

##### Acceptability of allocated treatment

The intervention leaflet was seen as informative, variously raising awareness of sitting time and the importance of PA (‘it’s a shock when you realise how long you actually sit’; Lincs, intervention, ID03), reminding participants of exercises they had not done for some time or suggesting new ways to be active in everyday settings. Several intervention recipients reported having recommended the leaflet to others. Some, however, felt that the intervention leaflet alone was insufficient to stimulate behaviour change and would benefit from endorsement from physicians.

Some wanted more compelling, physiological evidence for the benefits of the recommended activities, ‘to prove that they are actually useful’ (London, intervention, ID229), while some felt the leaflet was less applicable to them given their current levels of activity (‘a lot of my time is spent … hoovering or doing the housework … and none of that is mentioned’; London, intervention, ID136). The illustrations within the intervention leaflet were valued by many (‘the graphics … clearly clarified what you should be doing’; Kent, intervention, ID007), though some felt unable to identify with people depicted in the photographs (‘they’re a bit old’; London, intervention, ID136; ‘I would have had a few more different nationalities on the front, it’s … too white’; Kent, intervention, ID012).

Several participants thought the control factsheet was a useful reminder of the importance of exercise. Some felt, however, that it was ‘rather densely packed with information’ (London, control, ID001) and unclear, with no explicit definitions of exercise intensities. Others felt the recommendations lacked specificity, though many nonetheless attempted to adhere to them (‘I would have liked some specific suggested exercises … I tried to get vigorous exercise, but [with] some special structured exercises I would have done even better’; Kent, control, ID006). Many felt it lacked novelty (‘I’d be reluctant to give it to most of my friends, it would be a little insulting … they already know some of these things’; Lincs, control, ID004).

### Secondary outcomes: behaviour and habit

#### Validation of self-report data

Among participants who wore activPAL devices, comparisons with objective accelerometry offered mixed support for the validity of self-report data. Small- to medium-sized positive associations were typically observed between objective sedentary and self-reported sitting time, with correlations ranging from *r* = .07 to r = .47 [46]. Associations between step count and self-reported walking and moderate PA (correlation coefficient [*r*] range: −.15–.61), and stepping time and walking and moderate PA (*r* range: −.15–.60), ranged from small negative- to medium-positive associations. Negligible associations were observed between step count or time and vigorous PA (*r* range: −.15–.01; Additional file 5: Table S5).

#### Changes in behaviour and habit

Both intervention and control groups reported notable decreases in SB using the IPAQ measure, and, to a lesser degree, using the MOST measure (Table 3). The intervention group reported increases in walking but the control group did not. Both groups reported increases in moderate and vigorous PA and PA habit between baseline and 12 weeks, with greater apparent increases in the control group. Behavioural data did not therefore meet our criterion for direct progression to a full trial.

**Table 3:** Sedentary and physical activity behaviour and habit at baseline, 8 and 12 weeks, completers only

	Group	*N*	Baseline	8 weeks	12 weeks
			Mean (95% CI)	Mean (95% CI)	Mean (95% CI)
Sedentary behaviour					
Sitting time (IPAQ; min/day)	Intervention	35	501.49 (432.60, 576.68)	435.43 (381.59, 488.40)	408.43 (348.59, 471.99)
	Control	39	457.31 (117.99, 179.23)	426.03 (144.18, 209.22)	370.51 (317.74, 483.04)
Sitting time (MOST; min/day)	Intervention	44	565.05 (509.64, 626.04)	550.91 (492.73, 611.35)	550.57 (492.73, 611.35)
	Control	43	569.77 (504.89, 635.91)	541.16 (488.16, 595.33)	530.28 (461.25, 595.22)
SB habit	Intervention	44	4.09 (3.82, 4.36)	4.14 (3.82, 4.41)	3.95 (3.66, 4.20)
	Control	42	4.10 (3.88, 4.29)	3.90 (3.62, 4.21)	3.98 (3.64, 4.26)
Physical activity					
Walking (min/day)	Intervention	38	71.84 (51.34, 93.15)	84.92 (63.09, 113.42)	85.13 (57.65, 122.62)
	Control	40	114.88 (83.38, 149.12)	94.25 (72.51, 120.62)	101.75 (79.50, 126.38)
Moderate PA (min/day)	Intervention	37	33.65 (15.55, 57.15)	36.62 (20.81, 53.38)	34.59 (19.06, 53.51)
	Control	41	28.05 (12.80, 45.73)	46.95 (29.76, 63.78)	50.24 (28.42, 77.20)
Vigorous PA (min/day)	Intervention	43	4.07 (0.00, 10.35)	16.98 (6.98, 29.30)	15.35 (5.35, 28.13)
	Control	44	12.27 (3.41, 24.31)	21.84 (8.37, 38.06)	39.66 (18.75, 64.31)
PA habit	Intervention	44	3.25 (2.89, 3.59)	3.52 (3.16, 3.89)	3.66 (3.34, 3.95)
	Control	42	3.33 (3.02, 3.62)	3.57 (3.29, 3.81)	3.48 (3.14, 3.81)

*N* refers to sample size for within-group analyses using list-wise deletion

Few changes were observed in health and wellbeing, though both groups notably increased in leg strength (see Additional file 6: Table S6). Sensitivity analysis, accounting for non-completers, generated the same pattern of behaviour and habit changes (Additional file 7: Table S7). Within the control group, decreases in SB habit strength were moderately associated with decreases in SB assessed via the MOST (*ρ* = .43 [95% CIs: .13, .64]; Additional file 8: Table S8), but no such association was found in the intervention group (*ρ* = −.02 [95% CIs: −.31, .27]. All other relationships observed between SB or PA habit and behaviour change were small (*ρ* range: −.19–.16).

#### Qualitative analysis

##### Behavioural and health changes

Both intervention and control participants reported attempting to increase their PA (‘I’ve been walking more; I walk into town’; Kent, control, ID28), though some did so in anticipation of questionnaire completion and a perceived accountability to researchers:

I had to prepare for [the visits] and say, oh, today is the day [when my physical activity is] going to count. (London, intervention, ID212)

Many intervention recipients reported that adherence to the recommended activities became habitual and less effortful:

I’m [no longer] checking the booklet and making a list, [but] I am thinking ‘don’t use your hands when you get up. […] I am doing these little things throughout the day but not consciously. (Lincs, ID03)

Several obstacles to the habit formation process were reported. One intervention recipient participant reported difficulty in forming habit due to their unpredictable lifestyle (‘I did find it quite difficult to make it a sort of regular routine every day, because my days are all different’; Kent, ID001). For those participating in the trial over the 2014–15 Christmas and New Year period, the holiday period disrupted regular behaviour and contexts, making it harder to adhere to the tips.

Participants in both groups reported various health improvements, including improvements in functioning (‘I went out walking the other day and I was quite amazed at how quickly I was walking’; London, intervention, ID2136), physical fitness and sleep quality:

I never used to sleep properly at night, but since [participating in] this, the more exercise and the more activity I do before bed, I just get up once in the night and [sleep] straight until 6 in the morning. It’s much better. (London, intervention, ID291)

## Discussion

This study assessed the feasibility of trial procedures and explored the potential for a habit-based intervention to reduce SB and increase PA among older adults. Post-treatment attrition was low (7%), and although one adverse event occurred, the intervention recipient affected was motivated and able to complete the study. The intervention was generally viewed favourably, and mean adherence to all tips and all weeks was above 50%. However, behaviour change findings were mixed: while the intervention group self-reported reductions in SB, and increases in PA and PA habit strength, observed changes did not appear to be consistently greater than those in a control group, which received a non-habit-based informational factsheet outlining PA and SB health impacts and guidelines. Negligible impacts were found on health and wellbeing outcomes. The patterns of SB and PA observed in our sample suggest that we may have failed to recruit the most sedentary and inactive older adults, who may stand to benefit most from displacing SB with light PA [15].

Trial procedures met our criteria for progression to a definitive trial. Dropout (4/49 participants; 8%) was no higher than in the control condition (3/49; 6%) and was considerably lower than that observed in previous PA-promotion interventions (17%, albeit the median of 22 studies over a 6-month period [45]). Only one related adverse event was recorded. Qualitative data indicated that the intervention booklet was informative, and positive changes were reported in behaviour and health outcomes. Quantitative data indicated that the intervention group reduced total SB and increased PA and PA habit. These results were achieved via a self-administered and ‘lighter-touch’ intervention—i.e. an information leaflet supplemented by self-monitoring tick-sheets—than previous interventions, which have also shown promise for changing older adults’ SB and PA, but have predominantly involved objective monitoring and feedback, or one-to-one behavioural counselling [16–21]. However, behaviour changes were generally no more pronounced among intervention recipients than among the control group. The intervention group reported increases in walking where the control group did not, but the control group reported apparently greater increases in moderate and vigorous PA. Previous interventions have rarely been tested alongside minimal-treatment controls, so their relative effectiveness has not been estimated. A more realistic reading of our results is that our intervention, in its current form, has the potential to impact SB and PA, but to no greater extent than does an existing, non-theory-based treatment. We will therefore refine the intervention further prior to undertaking a definitive trial.

There are several potential explanations for the intervention conferring no apparent advantage over the control treatment in generating behavioural change. One is that the intervention was of limited effectiveness. It was designed to displace SB with PA, by pairing a ‘small changes’ approach with habit-formation principles, to promote integration of light PA into normally sedentary routines. Given mean adherence of over 50%, it is possible that our activity recommendations were too ‘light-touch’ to yield measurable changes in behaviour, or of insufficient instrumental value to be integrated into everyday settings. Indeed, more ostensibly functional tips (e.g. ‘leave the house daily’, weekly adherence range 64–80%) were apparently better adhered to than were less functional tips (e.g. ‘improve your posture’, range 31–35%).

Differences between groups may have influenced behavioural outcomes. At baseline, the control group was generally more physically active than the intervention group, reporting less sitting time (on the IPAQ index), and more walking and vigorous PA time. These differences, which are likely attributable to chance given random allocation to treatment conditions, may have distorted true treatment effects. It might be expected that higher baseline activity would have imposed a ceiling effect on activity gains in the control group, lessening the impact of the control treatment, and so, by comparison, enhancing the apparent effectiveness of the intervention treatment. However, it is also possible that the more active control participants may have been more receptive to novel strategies to increase activity, such as focusing on reducing sitting time. Our future trial will control for baseline differences between groups to control for such potential confounders.

Alternatively, study procedures may have influenced behaviour. Our active control treatment, selected to explore whether our intervention represents an improvement on an existing freely available intervention, may have suppressed intervention effects. Educating people of the dangers of SB and benefits of PA, and providing targets for PA frequency, duration and intensity, may perhaps be sufficient to change SB and PA, given low public awareness of the health detriments of SB [47–49]. Additionally, some of our participants valued the intervention simply as a reminder of the importance of PA [34], and the control treatment may also have served this purpose. Treatment effects may also have been obscured by self-reported PA and SB data, the accuracy of which has been questioned [50]. Comparisons with objective accelerometry data among a subsample showed inconsistencies in the accuracy of self-reports over time. True effects may have been affected by fluctuating levels of noise arising from unstable SB and PA recall errors. Although objective data could not be captured at most sites due to resource constraints, our findings testify to the importance of objective measures of SB and PA for intervention evaluation purposes. We will seek to use accelerometry data to more reliably evaluate the intervention in a future trial.

We may also have failed to recruit those for whom the intervention would have most effect. We originally intended to recruit people with ≥6 leisure time hours (360 min) sitting per day, and ≤30 consecutive minutes of leisure time activity of ≥3 metabolic equivalents per week [33]. Early recruitment experiences suggested that those self-declaredly meeting these criteria at consent had increased their PA above this threshold at baseline, so the inactivity criterion was removed [35]. Our sample self-reported, on average, 90 daily minutes of walking and 30 min of moderate PA at baseline, and a sizeable proportion of the sample reported less than 360 min of total SB on both indices at baseline. Our sample is thus unrepresentative of our intended target group of highly sedentary and inactive older adults. This may perhaps represent more favourable dispositions towards increasing PA and reducing SB among older adults who volunteered to take part in the study. Of 98 participants, 79 were recruited from three sites at which recruitment required eligible participants to initiate contact to express interest. This may have biased our sample towards more socially active older adults who tend to do more PA [51].

### Next steps

Our trial protocol appeared sufficiently feasible to form the basis of a larger, definitive trial. However, we may further refine our recruitment methods. Our intervention has not yet been evaluated among the most sedentary and inactive older adults, who may benefit most from displacing sedentary time with light PA [15]. We will seek to more effectively reach the most sedentary and inactive older adults. Our preceding uncontrolled trial, for example, showed that it is feasible to recruit from sheltered housing, residents of which are typically both highly sedentary and inactive [34, 52]. Further research might also explore whether our recommendations, or adaptations thereof, might be adopted for use among other populations characterized by SB and inactivity. An intervention aimed at reducing SB and promoting light PA may be of greater utility to the ‘oldest-old’ demographic (i.e. those aged 75+ years [53]) as a means of preserving physical functioning, rather than the 60–74 years demographic targeted in the present study. The PA and SB recommendations set out in our intervention have been incorporated into a SB reduction intervention for patients with COPD, a clinical population characterized by low levels of PA and high SB [54].

While the feasibility of our protocol warrants a larger trial, it would seem prudent to refine the intervention further prior to any further evaluation. Both the specific habit-based recommendations set out in our intervention, and the guidelines for PA and SB set out in the control treatment, showed potential for modifying SB and PA. While advising on appropriate PA and SB goals can potentially reduce SB [55], some participants felt that the control treatment could have been improved via the addition of more specific recommendations. A potentially fruitful next step may be to seek to incorporate elements of our habit-based approach, recommending integration of light PA into normally sedentary routines, into statements of PA and SB guidelines. This would produce a brief, self-administered and theory-based guidance document that not only advises on *which* behaviours people should adopt, but also *how* they may feasibly and sustainably do so, by making small adjustments to existing routines. Guidance on how to incorporate habit-formation techniques into brief advice is available [32, 56].

## Conclusions

Trial procedures were feasible, and our habit-based intervention was acceptable, though it appeared to yield no greater behavioural change than did a non-habit-based informational factsheet. Although intervention effects may have been suppressed due to high baseline PA and low SB levels among our sample, we will undertake further intervention development work prior to conducting a definitive trial. We will seek to explore the potential to combine elements of our intervention and to control treatments to enhance their effects and to more rigorously test a later iteration of the intervention among sedentary and inactive populations for whom changes in SB and PA would be most beneficial.

## Additional files

Additional file 1: Table S1. Intervention content: description and component behaviour change techniques. (DOCX 15 kb)

Additional file 2: Table S2. Baseline demographics, physical activity and sedentary behaviour: trial completers vs non-completers. (DOCX 17 kb)

Additional file 3: Table S3. Duration of research visits across sites and time points. (DOCX 13 kb)

Additional file 4: Table S4. Mean total per-week adherence to intervention tips, weeks 2–8 (*N* = 40, intervention group only). (DOCX 16 kb)

Additional file 5 **Table S5**. Correlations between objective and self-reported activity data. (DOCX 16 kb)

Additional file 6: Table S6. Health and wellbeing at baseline, 8 and 12 weeks, completers only. (DOCX 19 kb)

Additional file 7: Table S7. Behaviour, habit, health and wellbeing at baseline, 8 and 12 weeks, using baseline-observation-carried-forward imputation. (DOCX 20 kb)

Additional file 8: Table S8. Correlations between changes in habit and behaviour over 8 weeks by group, completers only. (DOCX 14 kb)

## Abbreviations

ANOVA: Analysis of variance
COPD: Chronic obstructive pulmonary disease
GP: General practitioner
ID: Identification code
IPAQ: International physical activity questionnaire
M: Mean
MOST: Measure of older adults’ sedentary time
NHS: National Health Service
PA: Physical activity
RCT: Randomised controlled trial
SB: Sedentary behaviour
SD: Standard deviation
UK: United Kingdom
W: Week

## Footnotes

1 Age criteria were imposed for two reasons. Firstly, age was used as a proxy for PA capability; the ‘young-old’ tend to have greater physical capacity for at least light intensity PA (roughly, those aged 60-74 [52]), relative to the ‘oldest-old’ (75+ years), for whom decline in physical functioning may impose limits on PA. Secondly, the ethics committee that reviewed procedures for the London site, originally the sole research site [35], stated that qualified health professionals would be required to screen participants aged 75y+ for suitability. We sought to standardise age eligibility criteria procedures across sites.

2 The same mailout company and postal service have been used, without problems, by colleagues in our research centre for previous studies using similar recruitment designs. Thus, we view these mailing errors as one-off, incidental risks, rather than a prevailing risk to be considered for any future definitive trial.
