# Supplementary material for: On Your Feet to Earn Your Seat: pilot RCT of a theory-based sedentary behaviour reduction intervention for older adults
Source: Pilot Feasibility Stud. 2017 May 8;3:23. doi: 10.1186/s40814-017-0139-6 (PMC5421328; doi:10.1186/s40814-017-0139-6)
Supplement: Supplementary file 1 — Intervention content: description and component behaviour change techniques. (DOCX 15 kb) [file 40814_2017_139_MOESM1_ESM.docx]

**Table S1.** Intervention content: description and component behaviour change techniques.

| *Booklet section* | *Informational content / behaviour change recommendations ** | *Specific PA forms targeted in tips* | *Behaviour change techniques* |
| --- | --- | --- | --- |
| Motivational text | - Regular PA in older age protects physical and mental health.  - PA includes aerobic, stretching, balance and strengthening.  - Sitting time is a risk factor for physical health.  - Limiting sitting to 20mins may protect health.  - Context-dependent repetition forms habit, which can maintain behaviour. | N/A | Information on health consequences; Framing/reframing;  Habit formation |
| Tips | “1. Leave the house daily: Ensure that you go out at least once a day. […] Don’t hesitate to use a stick if you need to.” | No explicit PA form, but conducive to standing (balance) and walking (aerobic) | Action planning;  Goal setting (behaviour);  Adding objects to the environment |
|  | “2. Make ad breaks active: When you watch TV, stand up or walk around during breaks between programmes. […] Try to watch TV for no more than one hour at a time, including two active breaks. Leave the remote control by the TV so that you have to get up to change channel.” | Standing (balance)  Walking (aerobic) | Prompts/cues;  Goal setting (behaviour);  Restructuring the physical environment;  Habit formation |
|  | “3. Take a stand: Stand up when waiting for a bus or train. Stay standing as long as possible. […] Make the sight of an empty seat a reminder to stand up.” | Standing (balance) | Prompts/cues;  Framing/reframing;  Habit formation;  Habit reversal |
|  | “4. Time to stretch: When sitting for long periods … set an alarm to go off every 20 minutes. When it rings, stand up and stretch, reaching your arms as high up as you can a few times. Hold each stretch for 10 seconds.” | Standing (balance)  Stretching (flexibility) | Prompts/cues;  Restructuring the physical environment;  Instruction on how to perform the behaviour;  Habit formation |
|  | “5. Rising and sinking: When standing by the sink in the kitchen … stand on your tip toes and then slowly drop back down onto your heels. Hold on to the counter if you need more support. Do this five times at first, and build up to 30.” | Standing (balance)  Stretching (flexibility) | Instruction on how to perform the behaviour;  Prompts/cues;  Habit formation |
|  | “6. Watch your step: Try to do at least 30 minutes of walking in total over the course of the day. This is equivalent to walking 1500 steps at a normal pace. You could start by aiming for 10 minutes (500 steps), and gradually build it up over time. […] Use a watch to time how long you spend walking. […] Look for opportunities to increase your steps, e.g.  – take the lift to one floor below your destination and walk up the last staircase;  - walk around your home when on the phone;  - park further away from the supermarket entrance;  - get off the bus a stop or two early;  - find a slightly longer route to get home;  - when meeting friends, go for a walk together rather than sitting down  - make more frequent shopping trips.” | Walking (aerobic) | Goal setting (behaviour);  Action planning;  Graded tasks;  Self-monitoring behaviour;  Behaviour substitution |
|  | “7. Sit to stand with no hands: Each time you stand up, try doing it without using your hands. Make sure your feet are flat on the floor and your chair is sturdy. […] As you get up, try holding your position a few inches above the chair and count to ten. You could also try standing up and then sitting back down again, gradually doing more as it becomes easier.” | Standing (balance)  Weight-bearing (muscle-strengthening) | Instruction on how to perform behaviour;  Graded tasks;  Habit formation |
|  | “8. Improve your posture: […] Stand with your back to the wall with your heels two inches from it. With your chin tucked in, move the back of your head towards the wall. Try to push the small of your back against the wall. Count to five, then repeat.” | Posture (flexibility) | Instruction on how to perform behaviour |
|  | “9. Limber up: Do these physical activities in the same order each morning, at your own pace:  9a. Calf stretch  9b. Chest stretch  9c. Walk as if on a tightrope across the floor  9d. March on the spot  9e. Walk your fingers up the wall  9f. Lift a tin of food in each hand.  Handy hint: Every evening, leave this booklet on your armchair or on the kitchen table to remind you to use it the next day.” ** | Stretching (flexibility)  Stretching (flexibility)  Stretching (flexibility)  Balance  Marching (aerobic)  Stretching (flexibility)  Weight-bearing (muscle-strengthening) | Instruction on how to perform behaviour;  Demonstration of behaviour;  Graded tasks;  Prompts/cues;  Restructuring the physical environment;  Habit formation |
|  | “10. Wall push-ups: do 10-push ups against a wall each morning. […] As your arms strengthen, increase the number of push-ups you do, resting for 1-2 minutes after every 10 push-ups.” | Weight-bearing (muscle-strengthening) | Instruction on how to perform behaviour;  Demonstration of behaviour;  Graded tasks |
| Habit-formation advice | Importance of:  - planning how, when and where to enact behaviour.  - increasing PA intensity gradually.  - repeating behaviour in stable settings  - continuing repetition after missed opportunities  - self-monitoring performance | N/A | Action planning;  Graded tasks;  Habit formation;  Self-monitoring behaviour |
| Misc advice | - Respond to urges to sit by reviewing whether “you have done enough to have earned that seat”, i.e. at least 10 consecutive minutes of PA.  - Monitor improvements in physical functioning arising from adherence to tips. | N/A | Framing/reframing;  Prompts/cues;  Goal setting (behaviour);  Self-monitoring outcome of behaviour |
| Supplementary tick-sheets | Daily self-monitoring record | N/A | Self-monitoring behaviour;  Self-monitoring outcome of behaviour |

* Tip descriptions are not comprehensive. Only text explicitly describing a behaviour change recommendation is included in this table; justifications or explanation of tips are not provided. ** Activities outlined in Tips 9a-9f and Tip 10 were outlined with extensive instructions and photographs, modelled by a female aged 66 years, to illustrate ergonomically correct procedures.
