# Supplementary material for: On Your Feet to Earn Your Seat: pilot RCT of a theory-based sedentary behaviour reduction intervention for older adults
Source: Pilot Feasibility Stud. 2017 May 8;3:23. doi: 10.1186/s40814-017-0139-6 (PMC5421328; doi:10.1186/s40814-017-0139-6)
Supplement: Supplementary file 2 — Baseline demographics, physical activity and sedentary behaviour: trial completers vs non-completers. (DOCX 17 kb) [file 40814_2017_139_MOESM2_ESM.docx]

**Table S2.** Baseline demographics, physical activity and sedentary behaviour: trial completers vs non-completers

| *Characteristics* |  | Completers  (N = 91) | Non-completers  (N = 7) |
| --- | --- | --- | --- |
| *Demographics* | | | |
| Gender: n (%) | *Available n* | *91* | *6* |
|  | Female | 53 (58%) | 4 (67%) |
|  |  |  |  |
| Age (years) | *Available n* | *89* | *6* |
|  | Mean (SD) | 68.36 (3.78) | 67.67 (4.08) |
|  |  |  |  |
| Ethnicity: n (%) | *Available n* | *88* | *6* |
|  | White | 85 (97%) | 6 (100%) |
|  | Black | 0 | 0 |
|  | Asian | 1 (1%) | 0 |
|  | Mixed or other | 2 (2%) | 0 |
|  |  |  |  |

| Marital Status: n (%) | *Available n* | *90* | *6* |
| --- | --- | --- | --- |
|  | Single | 7 (8%) | 0 |
|  | Married | 67 (74%) | 5 (83%) |
|  | Widowed | 8 (9%) | 1 (17%) |
|  | Divorced or separated | 8 (9%) | 0 |
|  |  |  |  |
| Longstanding illness: n (%) | *Available n* | *88* | *6* |
|  | Yes | 64 (72%) | 5 (83%) |
|  |  |  |  |
| Education, university: n (%) | *Available n* | *86* | *6* |
|  | Yes | 31 (36%) | 2 (33%) |
|  |  |  |  |
| Education, age leaving school (years) | *Available n* | *85* | *6* |
|  | Mean (SD) | 16.29 (1.39) | 17.00 (1.27) |
| *Sedentary behaviour* | | | |
| Sitting time (IPAQ; mins/day) | *Available n* | *84* | *6* |
|  | Mean (SD) | 477.23 (184.09) | 523.33 (238.72) |
|  |  |  |  |
| Sitting time (MOST; mins/day) | *Available n* | *90* | *6* |
|  | Mean (SD) | 556.97 (213.78) | 595.00 (234.24) |
|  |  |  |  |
| SB habit† | *Available n* | *89* | *6* |
|  | Mean (SD) | 4.10 (0.81) | 4.50 (0.55) |
| *Physical activity* | | | |
| Walking (mins/day) | *Available n* | *85* | *5* |
|  | Mean (SD) | 96.41 (92.02) | 62.00 (58.91) |
|  |  |  |  |
| Moderate PA (mins/day) | *Available n* | *89* | *6* |
|  | Mean (SD) | 33.54 (60.94) | 40.00 (64.81) |
|  |  |  |  |
| Vigorous PA (mins/day) | *Available n* | *90* | *6* |
|  | Mean (SD) | 9.28 (30.39) | 15.00 (36.74) |
|  |  |  |  |
| PA habit† | *Available n* | *89* | *6* |
|  | Mean (SD) | 3.30 (1.11) | 2.83 (1.47) |

Trial completers are those who completed all research visits (baseline, 8 and 12 weeks); non-completers are those who dropped out of the trial at some point after baseline. †Habit measured on a 1-5 scale, where 1= weak or no habit, and 5= strongest habit.
