# Supplementary material for: On Your Feet to Earn Your Seat: pilot RCT of a theory-based sedentary behaviour reduction intervention for older adults
Source: Pilot Feasibility Stud. 2017 May 8;3:23. doi: 10.1186/s40814-017-0139-6 (PMC5421328; doi:10.1186/s40814-017-0139-6)
Supplement: Supplementary file 3 — Duration of research visits across sites and time points. (DOCX 13 kb) [file 40814_2017_139_MOESM3_ESM.docx]

**Table S3.** Duration of research visits across sites and timepoints

|  |  | *Site* | | | |
| --- | --- | --- | --- | --- | --- |
| *Timepoint* |  | London | Lincs | Surrey | Kent |
| Baseline  (N = 84) | *N* | *20* | *3* | *20* | *41* |
|  | *Mean duration, mins (SD)* | 46.7  (14.4) | 20.0  (5.0) | 39.4  (10.3) | 19.7  (5.4) |
| 8-week follow-up  (N = 78) | *N* | *20* | *0* | *18* | *40* |
|  | *Mean duration, mins (SD)* | 23.0  (6.9) | - | 39.2  (11.5) | 20.9  (7.6) |
| 12-week follow-up  (N = 77) | *N* | *18* | *0* | *19* | *40* |
|  | *Mean duration, mins (SD)* | 40.4  (14.1) | - | 41.3  (16.9) | 31.6  (7.2) |

Ns vary due to missing data.
