# Supplementary material for: On Your Feet to Earn Your Seat: pilot RCT of a theory-based sedentary behaviour reduction intervention for older adults
Source: Pilot Feasibility Stud. 2017 May 8;3:23. doi: 10.1186/s40814-017-0139-6 (PMC5421328; doi:10.1186/s40814-017-0139-6)
Supplement: Supplementary file 4 — Mean total per-week adherence to intervention tips, weeks 2–8 (N = 40, intervention group only). (DOCX 16 kb) [file 40814_2017_139_MOESM4_ESM.docx]

**Table S4.** Mean total per-week adherence to intervention tips, Weeks 2-8 (N = 39, intervention group only)

| *Tips* | *Week* | *Mean adherence*  *(95% CI)* |
| --- | --- | --- |
| “1. Leave the house daily: Ensure that you go out at least once a day.” | Week (W)2 | 75.09% (64.10, 83.88) |
|  | W3 | 79.85% (69.24, 88.64) |
|  | W4 | 76.92% (65.93, 86.80) |
|  | W5 | 77.29% (65.93, 86.45) |
|  | W6 | 73.99% (62.27, 84.24) |
|  | W7 | 72.53% (60.44, 83.15) |
|  | W8 | 65.57% (54.58, 76.55) |
| “2. Make ad breaks active: When you watch TV, stand up or walk around during breaks between programmes.” | W2 | 58.97% (46.15, 70.33) |
|  | W3 | 54.58% (40.66, 66.67) |
|  | W4 | 50.55% (35.16, 62.99) |
|  | W5 | 49.45% (35.17, 62.27) |
|  | W6 | 47.62% (32.61, 61.17) |
|  | W7 | 46.15% (32.23, 58.97) |
|  | W8 | 42.12% (28.57, 55.68) |
| “3. Take a stand: Stand up when waiting for a bus or train.” | W2 | 58.24% (45.43, 69.23) |
|  | W3 | 56.41% (43.97, 68.13) |
|  | W4 | 57.51% (44.33, 71.05) |
|  | W5 | 53.11% (39.56, 67.03) |
|  | W6 | 49.45% (35.16, 65.20) |
|  | W7 | 51.28% (37.74, 65.19) |
|  | W8 | 38.46% (25.64, 53.11) |
| “4. Time to stretch: When sitting for long periods … set an alarm to go off every 20 minutes. When it rings, stand up and stretch … as high up as you can at least five times.” | W2 | 51.28% (37.73, 63.74) |
|  | W3 | 55.31% (41.40, 67.77) |
|  | W4 | 55.68% (41.76, 68.13) |
|  | W5 | 46.89% (31.87, 60.43) |
|  | W6 | 41.76% (28.21, 55.31) |
|  | W7 | 47.99% (34.80, 61.53) |
|  | W8 | 42.12% (28.21, 56.04) |
| “5. Rising and sinking: When standing by the sink in the kitchen … stand on your tip toes and then slowly drop back down onto your heels. Do this five times, building up to 30.” | W2 | 64.84% (52.01, 76.92) |
|  | W3 | 72.53% (60.81, 82.04) |
|  | W4 | 64.47% (52.75, 75.82) |
|  | W5 | 67.40% (53.11, 79.11) |
|  | W6 | 63.74% (50.55, 75.46) |
|  | W7 | 58.97% (46.15, 71.06) |
|  | W8 | 50.92% (38.10, 63.00) |
| “6. Watch your step: Try to do at least 30 minutes of walking in total over the course of the day.” | W2 | 57.88% (44.69, 69.60) |
|  | W3 | 57.88% (45.43, 70.69) |
|  | W4 | 62.64% (50.18, 75.08) |
|  | W5 | 57.51% (43.59, 71.42) |
|  | W6 | 60.81% (46.90, 73.62) |
|  | W7 | 64.84% (50.92, 76.56) |
|  | W8 | 54.58% (41.39, 67.40) |
| “7. Sit to stand with no hands: Each time you stand up, try doing it without using your hands.” | W2 | 69.96% (58.24, 81.32) |
|  | W3 | 66.67% (53.85, 78.02) |
|  | W4 | 69.60% (58.61, 80.22) |
|  | W5 | 68.13% (56.05, 79.11) |
|  | W6 | 68.50% (56.05, 79.11) |
|  | W7 | 62.64% (49.83, 75.09) |
|  | W8 | 49.08% (36.26, 62.27) |
| “8. Improve your posture: Stand with your back to the wall with your heels two inches from it … and move the back of your head towards the wall.” | W2 | 33.70% (21.98, 45.05) |
|  | W3 | 28.57% (17.22, 40.29) |
|  | W4 | 32.23% (19.06, 46.14) |
|  | W5 | 33.33% (20.51, 46.89) |
|  | W6 | 29.67% (17.22, 42.86) |
|  | W7 | 29.30% (16.86, 41.39) |
|  | W8 | 35.16% (21.61, 49.08) |
| “9. Limber up: |  |  |
| 9a. Calf stretch | W2 | 56.78% (42.87, 69.23) |
|  | W3 | 60.44% (47.62, 73.25) |
|  | W4 | 60.81% (47.62, 73.25) |
|  | W5 | 57.88% (43.96, 70.70) |
|  | W6 | 56.78% (42.49, 70.70) |
|  | W7 | 56.41% (41.40, 69.96) |
|  | W8 | 53.11% (38.10, 66.30) |
| 9b. Chest stretch | W2 | 52.01% (38.46, 65.19) |
|  | W3 | 57.51% (43.22, 69.96) |
|  | W4 | 55.31% (41.39, 68.13) |
|  | W5 | 56.41% (42.86, 70.33) |
|  | W6 | 51.65% (37.00, 65.57) |
|  | W7 | 53.85% (39.19, 67.40) |
|  | W8 | 46.89% (32.97, 60.44) |
| 9c. Walk as if on a tightrope across the floor | W2 | 38.83% (26.38, 52.74) |
|  | W3 | 39.19% (26.01, 53.11) |
|  | W4 | 42.49% (27.84, 57.13) |
|  | W5 | 40.66% (26.38, 55.31) |
|  | W6 | 45.42% (30.77, 59.71) |
|  | W7 | 38.10% (23.81, 52.74) |
|  | W8 | 32.97% (19.41, 46.89) |
| 9d. March on the spot | W2 | 52.01% (40.66, 63.74) |
|  | W3 | 56.04% (43.97, 68.50) |
|  | W4 | 58.61% (46.89, 70.33) |
|  | W5 | 62.27% (50.18, 74.35) |
|  | W6 | 63.00% (49.45, 76.56) |
|  | W7 | 53.48% (40.29, 66.30) |
|  | W8 | 52.75% (39.57, 65.20) |
| 9e. Walk your fingers up the wall | W2 | 37.00% (24.54, 49.45) |
|  | W3 | 39.93% (26.74, 52.75) |
|  | W4 | 39.93% (26.74, 53.48) |
|  | W5 | 39.19% (26.01, 53.11) |
|  | W6 | 41.39% (27.47, 54.95) |
|  | W7 | 35.90% (21.98, 49.45) |
|  | W8 | 36.26% (22.34, 49.82) |
| 9f. Lift a tin of food in each hand.” | W2 | 35.16% (22.34, 48.35) |
|  | W3 | 37.73% (24.18, 51.28) |
|  | W4 | 41.76% (28.21, 54.94) |
|  | W5 | 38.83% (24.19, 52.01) |
|  | W6 | 43.59% (29.67, 58.97) |
|  | W7 | 37.36% (22.35, 50.92) |
|  | W8 | 39.93% (26.37, 53.47) |
| “10. Wall push-ups: do 10-push ups against a wall each morning.” | W2 | 53.11% (39.93, 66.29) |
|  | W3 | 58.61% (46.15, 70.70) |
|  | W4 | 60.44% (47.62, 72.16) |
|  | W5 | 54.95% (43.22, 68.13) |
|  | W6 | 61.54% (47.62, 75.46) |
|  | W7 | 50.18% (35.54, 64.47) |
|  | W8 | 49.08% (34.80, 62.64) |

SD = Standard deviation, W = week number. Range for all tips: 0-100%.
