# Supplementary material for: On Your Feet to Earn Your Seat: pilot RCT of a theory-based sedentary behaviour reduction intervention for older adults
Source: Pilot Feasibility Stud. 2017 May 8;3:23. doi: 10.1186/s40814-017-0139-6 (PMC5421328; doi:10.1186/s40814-017-0139-6)
Supplement: Supplementary file 5 — Table S5. Correlations between objective and self-reported activity data. (DOCX 16 kb) [file 40814_2017_139_MOESM5_ESM.docx]

**Table S5.** Correlations (with 95% CIs) between objective and self-reported activity data

|  | Baseline (N = 16) | | | | 8 weeks (N = 14) | | | | 12 weeks (N = 16) | | | |
| --- | --- | --- | --- | --- | --- | --- | --- | --- | --- | --- | --- | --- |
|  | *Objective* | | | | *Objective* | | | | *Objective* | | | |
|  |  | SB (mins) | Step count | Stepping time (mins) |  | SB (mins) | Step count | Stepping time (mins) |  | SB (mins) | Step count | Stepping time (mins) |
|  | *Mean (SD)* | *602.94*  *(68.14)* | *7820.61*  *(2083.93)* | *101.21*  *(22.85)* | *Mean (SD)* | *618.09*  *(92.52)* | *7145.98*  *(2517.76)* | *92.25*  *(29.46)* | *Mean (SD)* | *604.63*  *(80.31)* | *7129.70*  *(2986.45)* | *94.47*  *(37.68)* |
| *Self-reported* |  |  |  |  |  |  |  |  |  |  |  |  |
| Sitting time (IPAQ; mins/day) | *444.81*  *(163.94)* | .32  (-.09, .63) | .11  (-.27, .56) | .12  (-.30, .58) | *446.07 (178.96)* | .19  (-.35, .69) | -.14  (-.80, .42) | -.07  (-.74, .54) | *427.19*  *(188.17)* | .28  (-.32, .73) | -.20  (-.65, .50) | -.22  (-.70, .52) |
| Sitting time (MOST; mins/day) | *561.38*  *(131.83)* | .45  (.10, .77) | -.20  (-.58, .28) | -.27  (-.69, .21) | *591.43*  *(167.60)* | .47  (-.11, .82) | -.19  (-.66, .27) | -.19  (-.70, .32) | *582.19*  *(190.82)* | .07  (-.49, .60) | .02  (-.49, .73) | .05  (-.48, .78) |
| Walking (mins/day) | *94.06*  *(64.63)* | -.03  (-.50, .49) | -.15  (-.65, .38) | -.15  (-.63, .44) | *80.36*  *(61.72)* | -.17  (-.66, .48) | .52  (-.40, .87) | .54  (-.36, .89) | *81.88*  *(35.11)* | -.58  (-.81, -.20) | .61  (.26, .89) | .60  (.24, .88) |
| Moderate PA (mins/day) | *40.94*  *(75.46)* | -.16  (-.51, .37) | .18  (-.30, .62) | .32  (-.27, .67) | *63.57*  *(57.49)* | -.09  (-.60, .56) | .26  (-.25, .70) | .33  (-.17, .80) | *60.63*  *(94.21)* | -.37  (-.73, .10) | .21  (-.26, .59) | .27  (-.26, .69) |
| Vigorous PA (mins/day) | *3.44*  *(9.44)* | -.33  (-.70, -.03) | -.03  (-.30, .27) | .01  (-.36, .29) | *12.86*  *(22.68)* | -.29  (-.73, .22) | -.15  (-.62, .47) | -.11  (-.55, .47) | *3.75*  *(15.00)* | -.10  (-.33, .04) | -.07  (-.24, .10) | -.10  (-.29, .03) |

CI: confidence interval, SB: sedentary behaviour.
