# Supplementary material for: On Your Feet to Earn Your Seat: pilot RCT of a theory-based sedentary behaviour reduction intervention for older adults
Source: Pilot Feasibility Stud. 2017 May 8;3:23. doi: 10.1186/s40814-017-0139-6 (PMC5421328; doi:10.1186/s40814-017-0139-6)
Supplement: Supplementary file 6 — Health and wellbeing at baseline, 8 and 12 weeks, completers only. (DOCX 19 kb) [file 40814_2017_139_MOESM6_ESM.docx]

**Table S6.** Health and wellbeing at baseline, 8 and 12 weeks, completers only

|  | *Range and interpretation (self-report measures)* | *Group* | *N* | *Baseline* | *8 weeks* | *12 weeks* |
| --- | --- | --- | --- | --- | --- | --- |
|  |  |  |  | *Mean*  *(95% CI)* | *Mean*  *(95% CI)* | *Mean*  *(95% CI)* |
| Confidence in balance | 1-3  (3: Greatest confidence) | Intervention | 44 | 2.70  (2.59, 2.80) | 2.73  (2.63, 2.83) | 2.71  (2.59, 2.82) |
|  |  | Control | 44 | 2.72  (2.58, 2.83) | 2.75  (2.64, 2.84) | 2.74  (2.63, 2.84) |
| Falls efficacy | 1-4  (4: Greater efficacy for avoiding falls) | Intervention | 37 | 3.56  (3.34, 3.75) | 3.64  (3.46, 3.79) | 3.71  (3.56, 3.83) |
|  |  | Control | 41 | 3.75  (3.62, 3.84) | 3.76  (3.61, 3.86) | 3.78  (3.64, 3.89) |
| Depression | 1-4  (4: Greater depression) | Intervention | 45 | 1.74  (1.50, 2.01) | 1.56  (1.37, 1.80) | 1.67  (1.43, 1.92) |
|  |  | Control | 43 | 1.64  (1.40, 1.92) | 1.56  (1.34, 1.81) | 1.59  (1.35, 1.90) |
| Pain | 1-6  (6: Most pain) | Intervention | 44 | 2.18  (1.91, 2.50) | 2.20  (1.93, 2.48) | 2.11  (1.86, 2.36) |
|  |  | Control | 43 | 2.21  (1.93, 2.47) | 2.16  (1.93, 2.40) | 2.28  (2.05, 2.51) |
| Stiffness | 1-5  (5: Most stiffness) | Intervention | 35 | 2.11  (1.86, 2.40) | 2.14  (1.89, 2.43) | 2.00  (1.71, 2.29) |
|  |  | Control | 34 | 2.26  (2.03, 2.53) | 2.06  (1.77, 2.32) | 2.26  (2.03, 2.53) |
| Quality of life | 0-100  (100: greatest quality of life) | Intervention | 39 | 74.96  (68.38, 81.15) | 75.03  (68.80, 80.67) | 74.92  (68.21, 81.08) |
|  |  | Control | 37 | 76.16  (71.60, 80.49) | 78.30  (73.78, 82.46) | 77.03  (71.81, 81.95) |

| Blood pressure (systolic) | - | Intervention | 38 | 134.24  (129.24, 140.29) | 135.71  (129.55, 142.34) | 135.24  (129.05, 141.00) |
| --- | --- | --- | --- | --- | --- | --- |
|  |  | Control | 44 | 142.32  (136.48, 148.81) | 140.18  (135.14, 145.68) | 140.91  (135.39, 146.95) |
| Blood pressure (diastolic) | - | Intervention | 38 | 75.87  (72.63, 78.97) | 77.61  (73.74, 81.82) | 77.66  (74.32, 81.11) |
|  |  | Control | 44 | 78.25  (75.18, 81.39) | 73.95  (71.11, 76.86) | 76.32  (73.43, 79.25) |
| Balance  (able to tandem-stand for 10s) | - | Intervention | Smallest N: 44 | Yes: N = 41  No: N = 4 | Yes: N = 41  No: N = 3 | Yes: N = 40  No: N = 4 |
|  |  | Control | Smallest N: 46 | Yes: N = 42  No: N = 4 | Yes: N = 41  No: N = 5 | Yes: N = 41  No: N = 5 |
| Leg strength  (able to rise from chair 5 times unaided) | - | Intervention | Smallest N: 44 | Yes: N = 40  No: N = 4 | Yes: N = 42  No: N = 2 | Yes: N = 40  No: N = 2 |
|  |  | Control | Smallest N: 45 | Yes: N = 44  No: N = 1 | Yes: N = 45  No: N = 1 | Yes: N = 45  No: N = 1 |
| Leg strength  (time to perform 5 chair rises) | - | Intervention | 40 | Time taken: 14.72  (13.62, 15.89) | Time taken: 13.42  (12.31, 14.65) | Time taken: 13.10  (12.14, 14.17) |
|  |  | Control | 44 | Time taken: 15.95  (13.76, 19.02) | Time taken: 12.71  (11.32, 14.33) | Time taken:  13.01  (11.30, 15.10) |
| Walking speed | - | Intervention | 43 | 3.05  (2.81, 3.37) | 2.83  (2.61, 3.07) | 2.85  (2.57, 3.21) |
|  |  | Control | 45 | 3.16  (2.81, 3.62) | 2.91  (2.65, 3.24) | 2.82  (2.57, 3.15) |

N refers to sample size for within-group analyses, using listwise deletion.

Description of objective measures:

*Blood pressure* represents second of two consecutive measurements captured by Omron M2 Classic monitors (Omron Healthcare, Kyoto, Japan); *balance* represents whether able to stand one foot directly in front of other for 10 seconds; *leg strength* measures assess whether able, and time taken, to rise from chair five times without using arms; *walking speed* represents faster of two consecutive 8ft walks.

Description of self-report measures:

*Quality of life* assessed using the EQ5D visual analogue scale [1]; *fear of falling* measured using the seven-item International Short Falls Efficiency Scale [2]; *confidence in balance* measured using the ten-item CONFBal scale [3]; *fatigue* measured two items from the CESD scale (‘Over the past week, how often did you feel [that everything you did was an effort/that you could not get going]?’; [4]); *pain* measured by a single item (‘Which of these best describes your pain over the last week?’ [1 = no pain; 7 = worst possible pain]; [5]); *stiffness* measured by a single item (‘Which of these best describes your general stiffness over the last week?’ [1 = no stiffness, 5 = very severe stiffness]).

**References**

[1] The EuroQol Group: **EuroQol - a new facility for the measurement of health-related quality of life.** *Health Policy* 1990, **16**:199–208.

[2] Geetrudis IJ, Kempen GI, Yardley L, van Haastregt JC, Zijlstra GA, Beyer N, Hauer K, Todd C: **The short FES-I: a shortened version of the falls efficacy scale - international to assess fear of falling.** *Age Ageing* 2008, **37**:45–50.

[3] Simpson JM, Worsfold C, Fisher KD, Valentine JD: **The CONFbal scale: a measure of balance confidence - a key outcome of rehabilitation.** *Physiotherapy* 2009, **95**:103–109.

[4] Radloff LS: **The CES-D scale: a self-report depression scale for research in the general population.** *Appl Psych Meas* 1977, **1**:385–401.

[5] Flaherty E: **Using pain-rating scales with older adults.** *Am J Nurs* 2008, **108**:40–47.
