# Supplementary material for: On Your Feet to Earn Your Seat: pilot RCT of a theory-based sedentary behaviour reduction intervention for older adults
Source: Pilot Feasibility Stud. 2017 May 8;3:23. doi: 10.1186/s40814-017-0139-6 (PMC5421328; doi:10.1186/s40814-017-0139-6)
Supplement: Supplementary file 7 — Behaviour, habit, health and wellbeing at baseline, 8 and 12 weeks, using baseline-observation-carried-forward imputation. (DOCX 20 kb) [file 40814_2017_139_MOESM7_ESM.docx]

**Table S7.** Behaviour, habit, health and wellbeing at baseline, 8 and 12 weeks, using baseline-observation-carried-forward imputation

|  | *Range and interpretation (self-report health and wellbeing measures only)* | *Group* | *N* | *Baseline* | *8 weeks* | *12 weeks* |
| --- | --- | --- | --- | --- | --- | --- |
|  |  |  |  | *Mean (95% CI)* | *Mean (95% CI)* | *Mean (95% CI)* |
| Sitting time (IPAQ; mins/day) |  | Intervention | 45 | 483.60  (422.95, 544.35) | 417.33  (368.23, 471.21) | 407.56  (354.45, 462.43) |
|  |  | Control | 45 | 477.00  (426.56, 523.10) | 429.89  (376.33, 485.54) | 399.11  (339.56, 457.99) |
| Sitting time (MOST; mins/day) |  | Intervention | 48 | 552.54  (495.36, 611.97) | 539.58  (478.03, 604.14) | 539.27  (474.27, 600.71) |
|  |  | Control | 48 | 566.15  (504.81, 627.91) | 543.75  (489.59, 596.03) | 540.35  (476.21, 603.60) |
| SB habit |  | Intervention | 47 | 4.11  (3.83, 4.36) | 4.15  (3.85, 4.40) | 3.98  (3.72, 4.21) |
|  |  | Control | 48 | 4.15  (3.92, 4.33) | 3.85  (3.54, 4.15) | 3.96  (3.65, 4.21) |
| Walking (mins/day) |  | Intervention | 46 | 78.67  (58.44, 98.55) | 89.71  (68.60, 113.66) | 73.11  (54.78, 92.21) |
|  |  | Control | 44 | 112.16  (82.62, 143.86) | 91.03  (69.21, 115.68) | 100.23  (78.41, 125.80) |
| Moderate PA (mins/day) |  | Intervention | 47 | 36.81  (20.96, 55.74) | 36.60  (22.23, 52.13) | 34.47  (19.79, 49.89) |
|  |  | Control | 48 | 31.15  (14.80, 50.42) | 47.92  (31.57, 68.43) | 52.92  (32.30, 78.23) |
| Vigorous PA (mins/day) |  | Intervention | 47 | 8.19  (1.81, 16.49) | 17.45  (8.30, 28.09) | 17.23  (6.81, 29.36) |
|  |  | Control | 49 | 11.02  (2.65, 21.42) | 20.22  (8.47, 36.47) | 37.35  (18.98, 58.77) |

| PA habit |  | Intervention | 47 | 3.30  (2.96, 3.66) | 3.55  (3.21, 3.87) | 3.68  (3.40, 3.96) |
| --- | --- | --- | --- | --- | --- | --- |
|  |  | Control | 48 | 3.25  (2.96, 3.56) | 3.48  (3.25, 3.73) | 3.35  (3.02, 3.69) |
| Confidence in balance | 1-3  (3: Greatest confidence) | Intervention | 48 | 2.71  (2.61, 2.81) | 2.74  (2.64, 2.84) | 2.72  (2.61, 2.83) |
|  |  | Control | 49 | 2.71  (2.58, 2.82) | 2.73  (2.62, 2.83) | 2.72  (2.62, 2.82) |
| Falls efficacy | 1-4  (4: Greater efficacy for avoiding falls) | Intervention | 42 | 3.60  (3.42, 3.76) | 3.67  (3.51, 3.80) | 3.73  (3.60, 3.84) |
|  |  | Control | 49 | 3.71  (3.60, 3.82) | 3.73  (3.59, 3.85) | 3.76  (3.64, 3.87) |
| Depression | 1-4  (4: Greater depression) | Intervention | 48 | 1.74  (1.51, 2.00) | 1.56  (1.36, 1.78) | 1.67  (1.44, 1.91) |
|  |  | Control | 49 | 1.64  (1.42, 1.90) | 1.59  (1.39, 1.82) | 1.60  (1.36, 1.88) |
| Pain | 1-6  (6: Most pain) | Intervention | 48 | 2.21  (1.94, 2.48) | 2.23  (1.98, 2.48) | 2.13  (1.88, 2.35) |
|  |  | Control | 49 | 2.24  (2.00, 2.53) | 2.12  (1.90, 2.35) | 2.27  (2.06, 2.49) |
| Stiffness | 1-5  (5: Most stiffness) | Intervention | 38 | 2.13  (1.84, 2.39) | 2.16  (1.89, 2.39) | 2.03  (1.74, 2.29) |
|  |  | Control | 38 | 2.26  (2.00, 2.53) | 2.08  (1.84, 2.34) | 2.29  (2.05, 2.53) |
| Quality of life | 0-100  (100: greatest quality of life) | Intervention | 45 | 75.07  (69.42, 80.13) | 75.11  (69.60, 79.87) | 74.69  (69.07, 79.98) |
|  |  | Control | 42 | 75.55  (70.86, 79.52) | 77.67  (73.17, 81.67) | 76.79  (71.55, 81.17) |

| Blood pressure (systolic) |  | Intervention | 42 | 135.00  (129.07, 140.76) | 136.33  (130.43, 142.76) | 135.90  (130.00, 141.74) |
| --- | --- | --- | --- | --- | --- | --- |
|  |  | Control | 47 | 141.68  (136.30, 147.17) | 140.06  (135.28, 145.27) | 140.74  (135.60, 146.25) |
| Blood pressure (diastolic) |  | Intervention | 42 | 76.19  (73.31, 79.19) | 77.76  (73.76, 81.62) | 77.81  (74.60, 81.24) |
|  |  | Control | 47 | 78.40  (75.47, 81.57) | 74.47  (71.64, 77.51) | 76.68  (73.96, 79.51) |
| Balance  (able to do tandem stand for 10s) |  | Intervention | Smallest N: 48 | Yes: N = 45  No: N = 4 | Yes: N = 45  No: N = 3 | Yes: N = 44  No: N = 4 |
|  |  | Control | 49 | Yes: N = 45  No: N = 4 | Yes: N = 44  No: N = 5 | Yes: N = 44  No: N = 5 |
| Leg strength:  able to rise from chair 5 times unaided? |  | Intervention | 48 | Yes: N = 43  No: N = 5 | Yes: N = 45  No: N = 3 | Yes: N = 45  No: N = 3 |
|  |  | Control | Smallest N: 48 | Yes: N = 47  No: N = 1 | Yes: N = 48  No: N = 1 | Yes: N = 48  No: N = 1 |
| Leg strength: time to perform 5 chair rises |  | Intervention | 45 | 14.89  (13.48, 16.41) | 13.70  (12.32, 15.24) | 13.00  (12.08, 14.05) |
|  |  | Control | 47 | 16.46  (14.34, 19.37) | 13.36  (11.81, 15.01) | 13.65  (11.86, 15.73) |
| Walking speed |  | Intervention | 48 | 3.09  (2.85, 3.37) | 2.91  (2.69, 3.12) | 2.91  (2.65, 3.22) |
|  |  | Control | 48 | 3.19  (2.88, 3.56) | 2.95  (2.70, 3.24) | 2.86  (2.62, 3.14) |

N refers to sample size for within-group analyses, using listwise deletion.
