# Supplementary material for: On Your Feet to Earn Your Seat: pilot RCT of a theory-based sedentary behaviour reduction intervention for older adults
Source: Pilot Feasibility Stud. 2017 May 8;3:23. doi: 10.1186/s40814-017-0139-6 (PMC5421328; doi:10.1186/s40814-017-0139-6)
Supplement: Supplementary file 8 — Correlations between changes in habit and behaviour over 8 weeks by group, completers only. (DOCX 14 kb) [file 40814_2017_139_MOESM8_ESM.docx]

**Table S8.** Correlations (95% CIs) between changes in habit and behaviour over 8 weeks by group, completers only

|  | *1.* | *2.* | *3.* | *4.* | *5.* | *6.* | *7.* |
| --- | --- | --- | --- | --- | --- | --- | --- |
| 1. SB habit change | - | .33  (-.003, .62) | -.22  (-.52, .13) | -.02  (-.31, .27) | .05  (-.27, .37) | -.19  (-.49, .15) | -.06  (-.36, .23) |
| 2. PA habit change | -.15  (-.44, .20) | - | -.10  (-.42, .24) | -.004  (-.32, .30) | -.07  (-.38, .29) | -.003  (-.33, .34) | .05  (-.27, .36) |
| 3. Sitting time (IPAQ; mins/day) | .11  (-.26, .47) | .02  (-.30, .36) | - | .53  (.19, .80) | -.20  (-.58, .17) | -.04  (-.42, .32) | .01  (-.28, .28) |
| 4. Sitting time (MOST; mins/day) | .43  (.13, .64) | -.19  (-.45, .12) | .55  (.26, .77) | - | .07  (-.27, .42) | -.01  (-.37, .34) | -.02  (-.31, .27) |
| 5. Walking change | <.001  (-.32, .30) | -.002  (-.32, .30) | -.10  (-.39, .22) | .13  (-.20, .44) | - | -.01  (-.43, .40) | .08  (-.27, .38) |
| 6. Moderate PA change | -.15  (-.41, .17) | .16  (-.17, .46) | -.30  (-.59, .08) | -.26  (-.54, .03) | .004  (-.32, .38) | - | .17  (-.31, .55) |
| 7. Vigorous PA change | -.15  (-.43, .15) | .11  (-.17, .36) | -.32  (-.59, -.03) | -.31  (-.60, .02) | -.25  (-.51, .05) | .12  (-.22, .44) | - |

Values are Spearman’s rho coefficients, due to non-normal distributions. N varies due to missing data. Values above the diagonal relate to intervention participants (N range: 38-45), values below the diagonal to control participants (N range: 41-45).
